# Predictors of nursing workers’ intention to leave the work unit, health institution and profession*

**DOI:** 10.1590/1518-8345.3280.3219

**Published:** 2019-12-05

**Authors:** Maiara Bordignon, Maria Inês Monteiro

**Affiliations:** 1 Universidade Federal da Fronteira Sul, Curso de Enfermagem, Chapecó, SC, Brazil.; 2 Scholarship holder at the Fundação de Amparo à Pesquisa do Estado de São Paulo (FAPESP) - Grant # 2016/06128-7, at the Conselho Nacional de Desenvolvimento Científico e Tecnológico (CNPq) - Grant # 162825/2014-5 and at the Coordenação de Aperfeiçoamento de Pessoal de Nível Superior (CAPES) - Grant # 01-P-3481/2014, Brazil.; 3 Universidade Estadual de Campinas, Faculdade de Enfermagem, Campinas, SP, Brazil.

**Keywords:** Intention, Employment, Occupations, Nursing, Nurses, Workplace Violence, Intenção, Emprego, Ocupações, Enfermagem, Enfermeiras e Enfermeiros, Violência no Trabalho, Intención, Empleo, Ocupaciones, Enfermería, Enfermeros, Violencia Laboral

## Abstract

**Objective:**

to identify the factors related to the nursing workers’ intention to leave the work unit, health institution and profession.

**Method:**

cross-sectional study with quantitative approach was carried out with 267 nursing workers from seven emergency units in Brazil. For data collection, we used the Questionnaire of socio-demographic, life style and work and health aspects as well as the Work Ability Index, Workplace violence questionnaire, questions about intention to leave and the Turnover Intention Scale. The predictors of intentions to leave were evaluated through Poisson regression models.

**Results:**

workplace violence increased and better satisfaction with current job decreased the probability of greater intention to leave the unit, institution and profession. Better work ability decreased the probability of greater intention to leave the unit and profession. The more qualified workers and those who had been working in the institution longer was more likely to greater intention to leave the profession.

**Conclusion:**

promoting job satisfaction, work ability and a violence-free environment is possible to decrease the workers’ intention to leave the job or profession, but nursing managers need to understand the three phenomena of intention to quit individually for retention strategies.

## Abstract (pt)

**Objetivo:**

identificar os fatores relacionados à intenção de trabalhadores de enfermagem em deixar a unidade de trabalho, instituição de saúde e profissão.

**Método:**

estudo transversal, com abordagem quantitativa, realizado com 267 trabalhadores de enfermagem de sete unidades de emergência no Brasil. Para coleta de dados, utilizou-se o Questionário de aspectos sociodemográficos, estilo de vida, trabalho e saúde, bem como o Índice de Capacidade para o Trabalho, questionário sobre violência no local de trabalho, questões sobre intenção em deixar e a Escala de Intenção de Rotatividade. Os preditores de intenção em deixar foram avaliados por meio de modelos de regressão de Poisson.

**Resultados:**

violência no trabalho aumentou e melhor satisfação com o emprego atual diminuiu a probabilidade de maior intenção em deixar a unidade, instituição e profissão. Melhor capacidade para o trabalho diminuiu a probabilidade de maior intenção em deixar a unidade e profissão. Os trabalhadores mais qualificados e aqueles que trabalhavam na instituição há mais tempo estavam mais propensos a maior intenção em deixar a profissão.

**Conclusão:**

com a promoção da satisfação no trabalho, da capacidade para o trabalho e de um ambiente livre de violência é possível diminuir a intenção de trabalhadores em deixar o emprego ou profissão, mas os gerentes de enfermagem precisam compreender os três fenômenos de intenção em sair individualmente para propor estratégias de retenção.

## Abstract (es)

**Objetivo:**

identificar los factores relacionados con la intención de los trabajadores de enfermería de dejar la unidad de trabajo, la institución de salud y la profesión.

**Método:**

se realizó un estudio transversal con enfoque cuantitativo con 267 trabajadores de enfermería de siete unidades de emergencia en Brasil. Para la recopilación de datos, utilizamos el Cuestionario de aspectos sociodemográficos, de estilo de vida y de trabajo y salud, así como el Índice de Capacidad para el Trabajo, el Cuestionario de violencia en el trabajo, las preguntas sobre la intención de dejar y la Escala de Intención de Rotación. Los predictores de intenciones de dejar fueron evaluados a través de modelos de regresión de Poisson.

**Resultados:**

la violencia en el trabajo aumentó y una mejor satisfacción con el trabajo actual disminuyó la probabilidad de una mayor intención de dejar la unidad, la institución y la profesión. Una mejor capacidad para el trabajo disminuyó la probabilidad de una mayor intención de dejar la unidad y la profesión. Los trabajadores más calificados y aquellos que han trabajado en la institución por más tiempo tenían más probabilidades de una mayor intención de dejar la profesión.

**Conclusión:**

promoviendo la satisfacción laboral, la capacidad para el trabajo y un ambiente libre de violencia es posible disminuir la intención de los trabajadores de dejar el trabajo o la profesión, pero los gerentes de enfermería deben comprender los tres fenómenos de intención de dejar individualmente para estrategias de retención.

## Introduction

The shortage of nursing workers is a global phenomenon and source of concern for several countries currently. The aging of the general population and of the nursing workforce coupled with the increase in demand for health care, decrease in interest for the nursing career and the desire of young nurses to leave their profession are some of the contributing factors resulting in the current shortage of nursing professionals^(^ 1 ^-^ 8 ^)^. These factors constitute the real nursing shortage, and there are authors who highlight the possibility of a pseudo-shortage, in which the number of nursing workers is sufficient, but they are not willing to work under the conditions offered by some health organizations^(^ 7 ^,^ 9 ^)^.

However, the nursing workers shortage is a challenge for health systems with regard to the ability to care for people, standards of care quality provided and overall health reach^(^ 1 ^,^ 10 ^-^ 12 ^)^, this is because nursing is responsible for most of the care provided on health services^(^ 7 ^)^. Studies indicate that the inadequate number of nurses can contribute to negative outcomes, such as patient mortality, infection and adverse events^(^ 7 ^,^ 13 ^-^ 14 ^)^. In addition, the reduction of nursing staff makes the remaining workers responsible for caring for a higher number of patients, increasing the workload and stress of the remaining workers and contributing to job dissatisfaction and burnout^(^ 7 ^,^ 10 ^,^ 15 ^-^ 16 ^)^.

For these reasons, the nursing workers retention is a particularly important issue for the health system, and initially requires an understanding of the factors that are associated with workers’ intention to leave their job and profession in each country^(^ 8 ^)^. As the factors are different among countries, it is relevant to analyze them within national contexts to support specific action plans^(^ 8 ^)^.

In Latin America, which includes Brazil, studies on nursing workers recruitment and retention are included in nursing research priorities^(^ 17 ^)^. Besides, estimates indicate that for each 100 young people there will be 153 elderly people in 2040 in Brazil, which requires health system preparation^(^ 18 ^)^. Nevertheless, the number of studies on the maintenance of a sufficient and specialized nursing workforce for health care, in order to avoid the lack of nursing workers in this country, is still restricted. These factors led us to carry out this study to identify the factors related to the nursing workers’ intention to leave the work unit, health institution and profession. The present study is one among the few studies that evaluated these three intentions to leave among Brazilian nursing workers.

## Method

This is a cross-sectional study with quantitative approach carried out with 267 nursing workers who worked in seven emergency units in three public health institutions in Brazil, with two institutions in the countryside of São Paulo state, and one in the countryside of Santa Catarina state. The work in emergency units includes caring for patients in complex conditions often unexpected and contact with anxious family members^(^ 19 ^)^. In addition, there is usually prolonged waiting time due to increased workload and high demands^(^ 19 ^-^ 20 ^)^. Emergency nursing workers are likely to situations that can lead to physical and mental exhaustion (e.g. workplace violence), besides contributing to the experienced nursing workers’ intention to leave their job or health institution^(^ 19 ^-^ 22 ^)^. The Research Ethics Board of our university approved this study (CAAE number: 41225814.4.0000.5404).

The participants were nurses, nursing technicians and assistants who are professional categories that integrate the nursing team in Brazil, and had been working in the units for at least three months, considering the importance of a work period for professional experience in the emergency unit and contact with their characteristics. We used a probabilistic sampling scheme that included stratification per work unit and professional categories, besides random sample of workers. According to the attributions of each professional within the nursing team, we defined two categories for the sampling scheme: (i) registered nurses and (ii) nursing technicians and assistants.

For sample calculation, we inserted an equal p proportion 0.50, significance level 5% and error 5%, which generated a minimum sample size of 201 nursing workers. We exceeded this minimum sample and collected data from 267 workers, ensuring a response rate of 63.7%. In order to ensure equivalence and strictly maintain the sampling scheme criteria, the number of participants that exceeded the minimum sample size was defined considering an additional proportion equivalent to the three health institutions. Finally, we distributed this number according to the amount of workers in each work unit and professional category. The workers which were not working (e.g. sick leave, holiday) did not participate in this study.

The data collection was carried out from 2015 to 2017. The workers were individually approached in their work units by the first author. Information about the research was provide and we invited them to participate voluntarily and anonymously. A consent form registered the workers’ agreement to participate of the study. The forms were returned to the researcher on the same invitation date, while the researcher remained in the unit to support or answer doubts, or at another agreed time between the parties. In this situation, the researcher returned to the unit in order to collect the forms.

For general data collection on the sample profile, we used the Questionnaire of socio-demographic, life style and work and health aspects (QSETS)^(^ 23 ^)^. This questionnaire was developed in Brazil and it has been used in different researches in occupational health area for two decades, including studies involving nursing workers^(^ 23 ^)^. The QSETS allowed variables collection such as: (i) socio-demographic variables - sex, age, marital status, having children and education level; (ii) lifestyle and life aspects variables - physical activity practice, satisfaction with current life, stress, tiredness and/or dismay after work, good sleep after work and number of leisure activities; (iii) health conditions - compared health and health problem in the last 15 days; and (iv) job-related features - professional category, time working in the work unit and health institution, age of first job and satisfaction with current job^(^ 23 ^)^.

We used the Turnover Intention Scale (EIR) to evaluate the worker’ intention to leave the health institution. This is a scale developed and validated in Brazil, which allows the worker to express to what extent he or she plans, thinks and desires to leave the institution. The response options ranged from one (never) to five points (always) and an average score was generated. The score classification ranged from high or medium intention (3 to 5 points) to low intention (1 to 2.9 points)^1^.

Two questions evaluated the nursing workers’ intention to leave the work unit and profession. The questions were: (i) Do you intend to quit working at the current emergency unit? (ii) Do you have intend to leave your profession? The workers responded in a response option scale with two extremes, zero (no intention) and ten (strong intention)^(^ 24 ^)^. For both intentions, we classified the scores into two categories: unintentional or low intention - score up to five points; and greater intention - score higher than five points.

For work ability evaluation, we applied the Work Ability Index (WAI)^(^ 25 ^)^. This index is originally Finnish and it was validated for use in Brazil^(^ 26 ^)^. The score ranges from seven to 49 points and indicates a better work ability as it increases^(^ 25 ^)^.

A questionnaire organized from previous measures and literature accessed the experience of workplace violence in the last 12 months, specifically three types of workplace violence: verbal abuse, sexual harassment and physical violence^(^ 27 ^)^. Five specialists in measures validation, workplace violence and nursing practice evaluated the questionnaire^(^ 27 ^)^. We considered victims of workplace violence who suffered at least one of three types of violence. In the same way, this criterion defined witnesses of workplace violence. In addition, these three types of violence supported the classification into analysis categories: not suffered, suffered one type, suffered two or all three types of violence, and had not witnessed, witnessed one type, witnessed two or all three types of workplace violence.

For data analysis, we used the Statistical Analysis for the Social Sciences (SPSS^®^, version 20.0) and Statistical Analysis System (SAS^®^, version 9.4). First, we analyzed the intention to leave the work unit, health institution and profession using descriptive statistics. Secondly, the chi-square test or Fisher’s exact test allowed us to analyze the existence of statistically significant associations between the intentions to leave and other categorical variables. The Mann-Whitney test and Kruskal-Wallis test evaluated differences between groups of intentions to leave regarding to numerical variables. The data did not adhere to the normal distribution from the Kolmogorov-Smirnov test and Shapiro-Wilk test, which supported the use of non-parametric tests. Finally, the analysis variables that resulted in p-value less than 0.20 were inserted as independent variables in the Poisson regression model corresponding to each intention to leave^(^ 28 ^-^ 29 ^)^. The independent variables insertion occurred in groups of similar variables (e.g. demographic variables groups - marital status and education level). The models structure respected steps and we inserted one variables group in each step^(^ 28 ^)^. The variables with statistical significance in the current analysis step remained in the model for analysis in the next step^(^ 28 ^)^. The variables sex, age and workplace adjusted the three models. We have always considered as significance level a p-value lower than 0.05.

## Results

In this study, 73% were nursing technicians or assistants and the others were nurses. The average age was 39.2 years (Standard Deviation - SD 9.9), ranging from 21 to 68 years. The average time working at the emergency unit was 5.5 years (SD 5.7) and 7.9 years (SD 7.4) at the health institution.

With respect to the nursing workers’ intention to leave the work unit the average was 2.9 (SD 3.4), ranging from zero to ten. 24.1% of workers expressed greater intention to leave the work unit. In Table 1, we presented the steps 6.1 and 6.2 that highlight the results of the Poisson regression model. There are two steps because they differ in relation to the insertion of workplace violence variables in two formats. In both steps, workers presenting perceptions that are more positive about their satisfaction with current job (neither satisfied, nor dissatisfied; satisfied or very satisfied) were less likely to manifest greater intention to leave the unit than dissatisfied or little satisfied workers. In this analysis, the probability of greater intention to leave was even lower in the group satisfied or very satisfied with job than among workers neither satisfied, nor dissatisfied (Table 1). In the present study, the Cronbach’s alpha for WAI was 0.70.

**Table 1:** Predictors of the nursing workers' intention to leave the work unit, Campinas, SP, Chapecó, SC, Brazil, 2015-2017 (n=220)*

		**Step 6.1**		
**Independent variables**	**PR^†^**	**Confidence interval (95%)**		**p-value**
		**Inferior bound**	**Upper bound**	
Satisfaction with current job (neither satisfied, nor dissatisfied)^‡^	0,55	0,34	0,88	0,0128
Satisfaction with current job (satisfied or very satisfied)^‡^	0,33	0,13	0,84	0,0208
Suffered one of three types of workplace violence^§^	2,71	1,29	5,69	0,0083
Suffered two or all three types of workplace violence^§^	2,29	1,03	5,09	0,0418
Work ability	0,93	0,90	0,97	0,0002
		**Step 6.2**		
**Independent variables**	**PR^†^**	**Confidence interval (95%)**		**p-value**
		**Inferior bound**	**Upper bound**	
Satisfaction with current job (neither satisfied, nor dissatisfied)^‡^	0,54	0,34	0,87	0,0111
Satisfaction with current job (satisfied or very satisfied)^‡^	0,33	0,13	0,86	0,0236
Suffered workplace violence (yes)||	2,58	1,24	5,37	0,0115
Work ability	0,93	0,90	0,97	0,0002

* Poisson regression model to estimate the probability of greater intention to leave the work unit (adjusted for age, sex and workplace), n = 220 considering missing data;

† PR = Prevalence ratio;

‡ Satisfaction with current job (dissatisfied or little satisfied) (reference);

§ How many types of workplace violence were suffered (did not suffer) (reference);

|| Suffered workplace violence (no) (reference)

Besides the satisfaction with current job, we found workplace violence and work ability as predictors of nursing workers’ intention to leave the unit. The self-reported victims of workplace violence were more likely to want to leave the unit than non-victims. For each point increased in the work ability score (better work ability), the probability of greater intention to leave decreased 7% (Table 1).

On the other hand, we found that 80.2% of nursing workers had low intention to leave the institution and 19.8% medium or high intention. The satisfaction with current job and workplace violence were also predictors of nursing workers’ intention to leave the institution, in both steps. The workers who were satisfied or very satisfied with their current job, neither satisfied/nor dissatisfied, were less likely to medium or high intention to leave than the group dissatisfied or little satisfied. In the self-reported victims of workplace violence, we identified greater probability of medium or high intention to leave the institution than non-victims. The probability of medium or high intention to leave the institution was 215% higher among victims of one type of workplace violence and 241% higher among workers who suffered two types or more, having as the reference group the non-victims (Table 2). In this study, the Cronbach’s alpha for EIR was 0.92.

**Table 2:** Predictors of the nursing workers' intention to leave the health institution, Campinas, SP, Chapecó, SC, Brazil, 2015-2017 (n=210)*

		**Step 6.1**		
**Independent variables**	**PR^†^**	**Confidence interval (95%)**		**p-value**
		**Inferior bound**	**Upper bound**	
Satisfaction with current job (neither satisfied, nor dissatisfied)^‡^	0,43	0,25	0,73	0,0019
Satisfaction with current job (satisfied or very satisfied)^‡^	0,20	0,05	0,83	0,0266
Suffered one of three types of workplace violence^§^	3,15	1,36	7,32	0,0076
Suffered two or all three types of workplace violence^§^	3,41	1,44	8,11	0,0055
Work ability	0,97	0,93	1,01	0,1286
		**Step 6.2**		
**Independent variables**	**PR^†^**	**Confidence interval (95%)**		**p-value**
		**Inferior bound**	**Upper bound**	
Satisfaction with current job (neither satisfied, nor dissatisfied)^‡^	0,43	0,25	0,74	0,0022
Satisfaction with current job (satisfied or very satisfied)^‡^	0,20	0,05	0,82	0,0259
Suffered workplace violence (yes)^||^	3,23	1,43	7,29	0,0047
Work ability	0,97	0,93	1,01	0,1272

* Poisson regression model to estimate the probability of medium or high intention to leave the health institution (adjusted for age, sex and workplace), n = 210 considering missing data;

† PR - Prevalence ratio;

‡ Satisfaction with current job (dissatisfied or little satisfied) (reference);

§ How many types of workplace violence were suffered (did not suffer) (reference);

|| Suffered workplace violence (no) (reference)

The intention to leave the profession was on average 2.2 (SD 3.4), ranging from zero to ten, and 17.3% of workers had greater intention to leave the profession. The satisfaction with current job, educational level, work ability and time working at the institution were independent variables related to intention to leave the profession in the two steps of the model. Data revealed that workers neither satisfied nor dissatisfied with current job were less likely to report greater intention to leave the profession than the dissatisfied or little satisfied group. The probability of greater intention to leave was even lower among satisfied or very satisfied workers with their current job, considering the same reference group. The probability of greater intention to leave the profession was higher among undergraduate and graduate workers in relation to the group up to technical level, and for each additional point in the work ability score, the probability of greater intention to leave decreased 5%. Moreover, for each additional point in the duration of work at the institution, the probability of greater intention to leave increased 8% (Table 3).

**Table 3:** Predictors of the nursing workers' intention to leave the profession, Campinas, SP, Chapecó, SC, Brazil, 2015-2017 (n=228)*

		**Step 5.1**		
**Independent variables**	**PR^†^**	**Confidence interval (95%)**		**p-value**
		**Inferior limit**	**Upper limit**	
Education level (undergraduate incomplete)^‡^	0,87	0,36	2,12	0,7638
Education level (undergraduate degree)^‡^	2,51	1,13	5,57	0,0242
Education level (graduate degree)^‡^	2,60	1,19	5,67	0,0161
Satisfaction with current job (neither satisfied, nor dissatisfied)^§^	0,35	0,20	0,61	0,0002
Satisfaction with current job (satisfied or very satisfied)^§^	0,07	0,01	0,44	0,0044
Time working at the institution (in years)	1,08	1,03	1,13	0,0011
Suffered one of three types of workplace violence^||^	1,32	0,73	2,38	0,3581
Suffered two or all three types of workplace violence^||^	2,05	1,03	4,08	0,0396
Work ability	0,95	0,91	0,99	0,0215
		**Step 5.2**		
**Independent variables**	**PR^†^**	**Confidence interval (95%)**		**p-value**
		**Inferior limit**	**Upper limit**	
Education level (undergraduate incomplete)^‡^	0,94	0,37	2,40	0,8957
Education level (undergraduate degree)^‡^	2,33	1,05	5,19	0,0375
Education level (graduate degree)^‡^	2,55	1,16	5,59	0,0196
Satisfaction with current job (neither satisfied, nor dissatisfied)^§^	0,38	0,21	0,68	0,0013
Satisfaction with current job (satisfied or very satisfied)^§^	0,07	0,01	0,43	0,0040
Time working at the institution (in years)	1,08	1,03	1,13	0,0028
Work ability	0,95	0,91	0,99	0,0108

* Poisson regression model to estimate the probability of greater intention to leave the profession (adjusted for age, sex and workplace), n = 228 considering missing data;

† PR - Prevalence ratio;

‡ Education level (until technical degree) (reference);

§ Satisfaction with current job (dissatisfied or little satisfied) (reference);

|| How many types of workplace violence were suffered (did not suffer) (reference)

Another predictor of nursing workers’ intention to leave the profession was the number of types of workplace violence suffered during the last year. The probability of greater intention to leave was 105% higher in the victims of two types or more of violence than among non-victims (Table 3).

## Discussion

In the literature, it is evident the importance of knowing the nursing workers’ intention to leave the unit, institution or profession and the factors that lead workers to want to leave the work and nursing^(^ 8 ^,^ 30 ^-^ 31 ^)^. In this way, we considered this recommendation and the need to expand studies on the nursing workers’ intention to leave the job and profession in Brazil to develop this research^(^ 32 ^)^, as well as the importance of contextual recognition for future design of actions focused on workers’ retention and quality care promotion^(^ 33 ^-^ 34 ^)^.

The results of the present study indicated that satisfaction with current job is a factor related with intention to leave the unit, institution and profession in Brazil. Other studies show similar results, highlighting the influence of job satisfaction on the worker’s intention to leave the job, position and field^(^ 21 ^-^ 22 ^,^ 31 ^-^ 32 ^,^ 35 ^-^ 37 ^)^. This fact suggests that promoting job satisfaction may be a possibility to decrease intention to leave and improve retention rates^(^ 21 ^-^ 22 ^,^ 31 ^-^ 32 ^,^ 35 ^-^ 37 ^)^. On the other hand, as this concept often involves more than one factor, to promote workers’ intention to remain working in the unit, institution or profession it will be necessary in some cases to know basis factors that lead to different levels of job satisfaction and which consequently contribute to the intention to leave^(^ 35 ^)^.

We found that nursing workers with higher qualification levels were more likely to express greater intention to leave the profession, agreeing with an integrative literature review on nurses’ intention to leave the profession^(^ 11 ^)^. The more qualified workers can represent a significant loss for the institutions, health system and nursing care. In addition, researchers revealed the relationship between nurse’s education and the probability of patients dying, indicating that an increase equal to 10% of nurses with bachelor in nursing decrease the patient deaths by 9%^(^ 14 ^)^. In this way, it is evident that education is one factor that can contribute to obtain better results in patient care^(^ 13 ^-^ 14 ^)^.

Furthermore, authors consider the elements of the work environment as special in the studies about the workers’ intention to leave^(^ 35 ^,^ 38 ^)^. The current study presented the workplace violence as a predictor of the three forms of intention to leave analyzed. We found that the probability of medium or high intention to leave the institution increased as the number of types of violence suffered was greater, but this result was not common for the intention to leave the unit, for example. We used a variable that represents the types of violence suffered and not the impact and number of times suffered, which may be behind this result^(^ 39 ^)^. At any rate, studies developed in several countries, such as England, China and Ghana, also identified correlations between workplace violence and workers’ intention to leave the institution and profession^(^ 21 ^,^ 30 ^-^ 31 ^,^ 40 ^)^. The results obtained coupled with previous researches suggest that promoting work conditions, good interpersonal relationships, safety and support of supervisors are strategies that can contribute to improve the retention of nursing workers^(^ 36 ^,^ 41 ^)^.

In the same way, the present study suggests that managers need to assess and promote the work ability in order to keep workers at the unit and profession^(^ 41 ^)^. This recommendation has support in the literature that confirms the influence of low work ability on the increase of intention to leave the unit, job and profession^(^ 41 ^-^ 42 ^)^. In this context, strategies aimed at building good interpersonal relationships are important because they can neutralize the impact of low work ability on the intention to leave^(^ 41 ^)^. The maintenance of work ability could also contribute to the retention of more experienced nursing workers who have a significant role for teaching, care and nursing management as a result of structured knowledge during their career^(^ 6 ^,^ 41 ^)^. However, the most experienced workers at the institution were more likely to have greater intention to leave the profession in our study. It is evident that managers need to provide attention to all these details and find alternatives for action while the workers have the intention, but have not yet definitely opted for leaving the institution and later, the nursing profession^(^ 11 ^,^ 33 ^,^ 43 ^-^ 44 ^)^.

This study has limitations. First, the cross-sectional study characteristics do not allow for the establishment of causal relationships, although they indicate the influence of certain variables on the outcomes of interest^(^ 43 ^)^. Secondly, we seek to ensure the use of questionnaires with known psychometric properties, but this was not possible in all cases. On the other hand, experts evaluated the questionnaire on workplace violence during the face validity process and the socio-demographic data questionnaire integrates several studies within occupational health area over the years. In Brazil, the low availability of validated measures to assess workplace violence is a challenge for researchers of this theme^(^ 27 ^)^. Thirdly, we defined the sample size in order to estimate the proportion of workplace violence, considering the central axis of our study and the existence of more than one outcome. Another study limitation is the evaluation of the previous events of workplace violence, which demanded the recall of participants. In these cases there is the possibility of memory bias, but the authors recognize that events are better remembered when marked by negative experiences or related to risk factors (e.g. workplace violence), and when participants are asked to provide details about it^(^ 45 ^)^.

This research has implications for health organizations and new studies. We recognize the relevance of studies on the nursing workers’ intention to leave the unit, institution and profession in Brazil, besides the identification of the strength of each factor in the intention to leave within specific groups such as professional categories and people in different phases of their career and life. The retention factors among younger workers and people working for more time may be different, and both groups are important for the performance of the health institution and system to work collectively.

In this context, it is interesting to highlight that not all workers who suffered workplace violence expressed intention to leave. Our theory proposes that the worker’s intention to leave and their effective departure, when not motivated by good new opportunities, is be related to the existence of a balance or imbalance in other domains (affective/emotional, family, financial or interpersonal relationship/social domain). The studies on life and professionals trajectories can be important in this sense and they need to articulate the career and life phases, as well as the individual and collective support network.

The permanence of workers when they are determined to leave the unit, institution or profession is another element that requires attention in terms of worker’s health, professional-patient/professional interaction and nursing care. The fact of working in an unwanted environment can be a highly negative experience for the worker’s health particularly when the regulation of emotions is fragile. In this way, to study the nursing workers’ intention to leave the unit, institution and profession is important in order to monitor the dynamics of intentions and subsequently act in advance to avoid crises in the Brazilian nursing workforce and in other countries.

## Conclusion

From this study, we conclude that workplace violence and satisfaction with current job are important factors for nursing workers’ retention because they predicted the three forms of intention to leave. Better work ability also decreased the probability of greater intention to leave the unit as well as the profession. In this way, we identified that some factors are common among the intention to leave the unit, institution and profession, but there are elements that relate only to one of these three forms. The results suggest that promoting job satisfaction, work ability and a safe environment in relation to workplace violence, it is possible to decrease the workers’ intention to leave the job or profession, but nursing managers need to understand the three phenomena individually to propose nursing workforce retention strategies according to the institution’s objectives or health system.

## Footnotes

1 Siqueira MMM, Gomide S Júnior, Oliveira AF, Polizzi A Filho. Intenção de rotatividade. In: Siqueira MMM (Org.) Novas medidas do comportamento organizacional: ferramentas de diagnóstico e de gestão. Porto Alegre: Artmed; 2014. p. 209-16.
